# Synovial Macrophages in Osteoarthritis: The Key to Understanding Pathogenesis?

**DOI:** 10.3389/fimmu.2021.678757

**Published:** 2021-06-15

**Authors:** Amanda Thomson, Catharien M. U. Hilkens

**Affiliations:** Immunotherapy Research Group, Translational and Clinical Research Institute, Faculty of Medical Sciences, Newcastle University, Newcastle Upon Tyne, United Kingdom

**Keywords:** osteoarthritis, pathogenesis, macrophage subsets, synovial tissue, inflammation

## Abstract

Effective treatment of osteoarthritis (OA) remains a huge clinical challenge despite major research efforts. Different tissues and cell-types within the joint contribute to disease pathogenesis, and there is great heterogeneity between patients in terms of clinical features, genetic characteristics and responses to treatment. Inflammation and the most abundant immune cell type within the joint, macrophages, have now been recognised as possible players in disease development and progression. Here we discuss recent findings on the involvement of synovial inflammation and particularly the role of synovial macrophages in OA pathogenesis. Understanding macrophage involvement may hold the key for improved OA treatments.

## Introduction

Osteoarthritis (OA) is the most common form of arthritis, characterised by pain, swelling and stiffness of the joint. It is also multifactorial in nature, with associated risk factors such as age, sex, ethnicity and obesity. Primary locations affected are synovial joints, including the knee, hip and hands, with knee OA being most frequently observed. OA affects 7% of the global population and it is estimated that one third of people over the age of 65 suffer with the disease. This equates to approximately 500 million individuals, a figure which has risen by 48% from 1990-2019 (1–3). Even though OA is a leading cause of disability, the 15^th^ highest cause of years lived with disability globally, no cure or disease modifying treatments are available (1). Symptoms are typically managed through a combination of non-pharmacological methods and non-steroidal anti-inflammatory drugs (NSAIDS). Surgical intervention through joint replacement still remains the only option for end-stage disease, emphasising the need for better treatment strategies. Here, we provide a short overview of the role of inflammation in OA pathogenesis, with a specific focus on the involvement of synovial macrophages. Unravelling the role of these cells may lead to improved stratification of OA patients for anti-inflammatory treatments and/or the identification of novel therapeutic targets.

## OA Pathogenesis: Cartilage and Subchondral Bone

OA can affect the entire joint, including cartilage, synovial tissue, subchondral bone and the joint capsule, as well as ligaments and periarticular muscles (4). Cartilage degeneration is probably the most well-known hallmark of OA, and many studies have focused on understanding and preventing its destruction (5). The cartilage provides an important lubricated covering to the bone surfaces where the femur, tibia and patella articulate with each other. This absorbs stress created during movement and importantly, creates a smooth platform to allow for efficient joint motions. It comprises an extracellular matrix (ECM), composed mostly of type II collagen and aggrecan proteoglycans, which bring strength and flexibility to the tissue respectively (6, 7).

Chondrocytes constitute the cellular component (7) and maintain homeostasis through synthesis and degradation of the cartilage proteins. In OA, this equilibrium shifts to catabolism and chondrocytes adopt an activated state characterised by increased cell proliferation, molecular alterations and production of ECM degrading enzymes. This leads to cartilage damage (8–10). MMP family proteins and aggrecanases, the most widely studied ECM degrading enzymes, are both able to degrade native collagen and aggrecan (11–13). This breakdown of the ECM leads to fibrillation and subsequent fissure development within the cartilage layers and as a result, the subchondral bone (SB), situated directly beneath, is exposed to the articular cavity. The joint therefore becomes unable to function normally with regards to gliding movements and is incapable of effectively absorbing mechanical stress. Composed of the SB plate and underlying trabecular and subarticular bone, the SB functions as a shock absorber and helps distribute the mechanical load of the joint. Structural changes to SB can also be seen in OA, and include increased bone turnover, the development of microfractures and increased angiogenesis. Bone sclerosis, osteophytes, bone cysts and bone marrow lesions, detected *via* MRI can also be seen.

Inflammatory mediators have long been known to play a role in the breakdown of the cartilage ECM. In particular, OA patients have increased levels of IL-1β, TNF-α and IL-6. IL-1β is an essential mediator of joint inflammation and its overexpression by chondrocytes can be seen in early osteoarthritic cartilage (14, 15). Such levels cause an abnormal chondrocyte phenotype, which directly interferes with the synthesis of ECM collagen and aggrecan proteins. An associated increased release of MMP and aggracanase enzymes such as MMP-1, MMP-3 and MMP-13 is also seen, with destructive effects on cartilage components (16, 17). Functioning in an autocrine manner, IL-1β can induce its own secretion and stimulate the synthesis of other inflammatory mediators, again such as TNF-α and IL-6. Often found working in synergy with IL-1β, TNF-α binding to its receptors induces a similar NF-κB signalling cascade to increase inflammation and catabolism through enhancing adhesion molecule expression, the synthesis of further cytokines, and promoting the expression of more MMP family enzymes able to degrade the ECM (18, 19). Other cytokines such as IL-8, IL-18, IL-17 and IL-22 are increased when comparing human inflamed and non-inflamed synovial tissue (20). In particular such cytokines are associated with Th17 and NK22 cells, along with the recruitment of neutrophils into the tissue, all of which are capable of further promoting synovitis (20). IL-6, produced by cells such as chondrocytes, osteoblasts, fibroblasts, macrophages and adipocytes can also synergise with other cytokines to affect the ECM. However, evidence indicates IL-6 is the key cytokine to affect the SB layer of the joint by promoting the formation of osteoclasts to increase bone absorption within the joint (21, 22). Also associated with the bone itself, high TGF-β has been reported *via in vitro* and *in vivo* studies to promote the production of osteophytes, as well as increasing chondrocyte hypertrophy *via* alternative signalling pathways (23). Further descriptions of cytokines involved in OA are beyond the scope of this mini review, but such information has been discussed elsewhere (24).

## OA Pathogenesis: Synovial Tissue

The synovium lines the joint cavity. Its main function is to produce synovial fluid to equip the joint for efficient movement. Concentrations of synovial fluid components (lubrican and hyaluronic acid) are often altered in OA, influencing cartilage integrity. The synovium is composed of two main regions: the lining and sublining layers. Synovial lining consists mainly of macrophage and fibroblast cell types. The sublining contains additional fibroblasts, macrophages, adipose cells and blood vessels, with low numbers of lymphocytes also detectable (25).

Low-grade synovial inflammation has been observed in over half of OA patients at both early and late stages of disease (26–33) which has led to the notion that OA is not simply caused by an age-related wear and tear of the joint. Unlike more typical inflammatory arthritides [e.g., rheumatoid arthritis (RA)] OA synovitis is usually not accompanied by overt systemic inflammation. In RA, the inflamed synovium is characterised by vasculitis and a mixed immune cell infiltrate. This infiltrate predominantly consists of lymphocytes but also includes myeloid cells such as macrophages. Inflammation and angiogenesis in RA are further exacerbated due to antigen presentation and cytokine release. Subsequently, cartilage degradation and bone erosion arises over time in response to protease (e.g. MMPs) and cytokine release (e.g. TNF-α and IL-6) (34). A role for macrophages, with their associated inflammatory cytokines (IL-6 and TNF-α), has also been recognised in RA (35).

In OA, synovial inflammation is less pronounced, but there is ample evidence to support its pathogenic role (26, 30, 36). Histopathological studies since the 1980s have identified inflammatory signatures (cellular hyperproliferation, increased angiogenesis and lymphocyte aggregate appearance) within OA synovium (37–39). The degree of inflammation is highly heterogenous between patients, but nevertheless, has been associated with pain and disease progression. As highlighted in the previous section, inflammatory molecules, including IL-1β and TNF-α, are able to induce protease secretion by chondrocytes, highlighting possible crosstalk between the synovium and other joint tissues (40). It has also been reported that the quantity of activated macrophages within patient OA synovium correlates with disease severity and progression (41). Synovitis confers a 9-fold greater risk of individuals presenting with painful knee OA (30). Elevated inflammatory markers have been detected in the serum and synovial fluid of OA patients and levels of serum TNF-α correlate with OA kellgren-lawrence X-ray grades (42). However, whilst treatments to dampen inflammation in RA have shown success (43), trials with anti-inflammatory drugs in OA have been disappointing thus far. A possible reason may be that the inflammatory players and processes differ greatly between patients; a notion that is supported by heterogeneity of the immune cell infiltrate in the OA synovium. For example, bi-compartmental OA is characterised by higher infiltration of CD4^+^ T cells into the synovium than uni-compartmental disease (44) and we recently demonstrated highly variable numbers of macrophages as well as other immune cell subsets in the OA synovium (45). Therefore, we argue that a better understanding of the inflammatory players in OA would benefit the development of improved therapeutic strategies; either through stratification of OA patients for the most suitable disease-modifying treatments and/or the identification of novel targets.

## Synovial Macrophages

**Figure 1:**
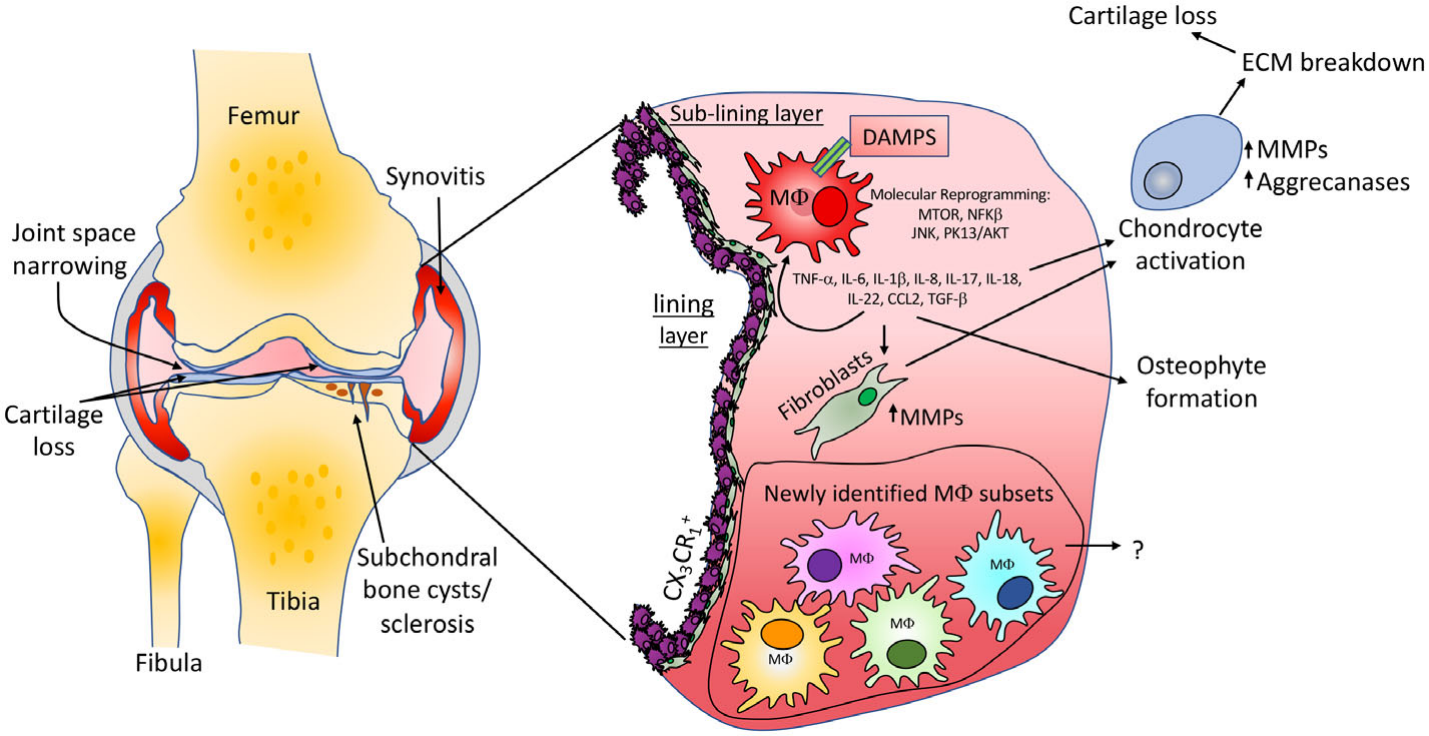
Knee osteoarthritis pathology and macrophage involvement. Common features of OA including cartilage loss, narrowing of the joint space, synovitis and the development of subchondral bone cysts and sclerosis are shown. Macrophages in the synovium can contribute to OA *via* the release of inflammatory molecules which are able to stimulate resident fibroblast populations to produce cartilage extracellular matrix degrading enzymes. Inflammatory molecules are also able to activate chondrocytes, promoting an abnormal molecular and cellular phenotype, again promoting cartilage loss. We suggest that the same or similar macrophage populations newly identified in inflammatory arthritis studies will be found in OA tissues, may differ between clinical states and could provide therapeutic targets for subgroups of patients. The identification and impact of such populations in OA development is yet to be determined.

The most abundant immune cell type in the OA synovium is the macrophage. Macrophages are often described as displaying an M1 or M2 phenotype. Activated by environmental factors such as IFN-γ, TNF-α and LPS, M1 macrophages secrete pro-inflammatory cytokines and low levels of IL-10. M2 macrophages display an anti-inflammatory profile and possess tissue-repair functions (46). However, such M1/M2 descriptions are now regarded as extreme poles of a spectrum in many fields, with macrophage phenotype varying greatly depending on the tissue environment. High macrophage numbers are detected in OA patients compared to healthy controls and quantities of activated macrophages correlate with clinical symptoms (41, 47). Furthermore, increases in macrophage associated molecules (sCD163 and sCD14) and chemoattractants such as CCL2 and CX_3_CL_1_ in OA patient synovial fluid are linked with clinical outcome in OA (48, 49). It is thought that synovial macrophages respond to danger-associated molecular associated patterns, including cartilage fragments and intracellular proteins from necrotic cells, consequently contributing to cartilage damage and bone alterations through the release of cytokines such as IL-1β, TNF-α and TGF-β (**Figure 1**). In support of this, *in vitro* studies have highlighted that depletion of CD14^+^ macrophages from synovial cell cultures results in a reduction of IL-1β, TNF-α, MMPs and aggrecanase enzymes which are able to degrade joint cartilage (50). Latest research investigating joint macrophages under normal and disease conditions (RA and OA) has led to the identification of multiple synovial macrophage subsets in the same joint. In a setting were inflammation aids disease, it’s probable that the abundance of distinct macrophage subsets could perpetuate or indeed help to resolve OA.

## Novel Synovial Macrophage Subsets in Inflammatory Arthritis

To carry out comprehensive investigations of joint macrophages Culemann and colleagues utilised transgenic mice and models of inflammatory arthritis (51). Exploring the origin of increased macrophage numbers during arthritis, the authors identified a CX_3_CR_1_+ macrophage subset in direct proximity with collagen VI expressing synovial fibroblast cells. This subset expressed tight junctional protein markers usually associated with that of endothelial cells (F11r, ZO-1 and claudin-5), was maintained by a distinct Ki67+ CX_3_CR_1_- interstitial macrophage population and formed a dense barrier between the synovial capillary network and the intra-articular space. At the onset of arthritis CX_3_CR_1_+ macrophages underwent altered morphology, and their cell-to-cell contacts were abrogated. Consequently, there was a breakdown of the “macrophage barrier” and CX_3_CR_1_- macrophage populations were found to rapidly proliferate in response. Coincidingly blood-derived macrophages infiltrated the tissue. The CX_3_CR_1_+ macrophage subset may therefore promote an important synovial regulatory function to seclude and protect intra-articular structures. Work to identify macrophage populations capable of promoting a “macrophage barrier” and the downstream implications of this in human OA could prove to be highly advantageous. Comparing RNA sequencing of mouse macrophage populations the authors also showed that CX_3_CR_1_+ macrophages expressed immune related genes (TREM2 and VSIG4) and that additional heterogeneity existed within CX_3_CR_1_- macrophages (51). This is a significant observation as mouse macrophage expression profiles correlate with recent sc-RNA sequencing data sets from human RA patients. Exploring human synovial macrophages within RA, and using OA tissue as a comparison, Zhang and colleagues identified four transcriptionally distinct subsets (SCM1-M4) (52). In particular, the presence of IL-1β+ pro-inflammatory macrophages (SCM1 subset) were upregulated in “leukocyte-rich” RA tissue compared to OA samples. Conversely, a SCM2 subset, which express VSIG4 similar to mouse CX_3_CR_1_+ macrophages, were upregulated in OA, suggesting they too are a resident synovial population.

In line with this, Alivernini and colleagues recently identified that clinically distinct states of RA can be characterised by relative proportions of particular macrophage populations. Firstly, healthy donor macrophages and those from patients in remission phenotypically were MerTK+ and CD206+. Patients with active RA had higher amounts of MerTK- CD206- and fewer MerTK+ CD206+ macrophages (53). Delving further to unravel the heterogeneity of these two populations, sc-RNA sequencing revealed nine distinct synovial macrophage clusters that could be classified into again four subpopulations: TREM2+, FOLR2^high^, HLA+ and CD48+. Comparing relative gene ontology pathways of the nine macrophage clusters with clinical state revealed that MerTK+ TREM2+ and MerTK+ FOLR2+ macrophages were predominantly in healthy tissue. They also showed gene expression (ALDH1A1 and VSIG4) that would promote regulation of adaptive immunity through inhibition of T effector cells. Patients with sustained remission showed an increase in MerTK+ FOLR2^high^ LYVE1+ macrophages which link this subset to tissue remodelling and homeostasis. In comparison, treatment naïve and active RA had increased proportions of MerTK- CD48- SPP1+ and MerTK- CD48- S100A12+ clusters which showed a pro-inflammatory transcriptome phenotype. The authors further showed that the MerTK- CD206- and MerTK+ CD206+ macrophage clusters were able to induce proinflammatory responses and repair synovial fibroblasts phenotypes, respectively. An overview of all novel synovial macrophages can be seen in **Table 1**.

**Table 1:** Recently Identified novel macrophage subsets.

Reference	Species	Macrophage subset	Sub-populations	Clusters identified	Associated disease or clinical state	Associated surface marker or gene expression	Location in Synovium	Additional information
(52)	Human	SC-M1: IL-1β^pos^, NR4A2^pos^ HBEGF^pos^ PLAUR^pos^ RGS2^pos^ ATF3^pos^	–	–	↑ leukocyte-rich RA	Mass cytometry: CD11C+ CD38+ RNA-seq: CD14+ CD11C+++ CD38+++	Not specified	–
		SC-M2: MerTK^pos^ HTRA1^pos^	–	–	↓ leukocyte-rich RA ↑ OA	Mass cytometry: CD11C- RNA-seq: CD14+ CD11C+ CD38-	Not specified	Possibly equivalent to mouse resident macrophage populations.
		SC-M3: CD14^pos^ C1QA^pos^ MARCO^pos^	–	–	Marginally ↑ OA	–	Not specified	Possibly equivalent to mouse resident macrophage populations.
		SC-M4: LY6E^pos^ IFITM3^pos^ IFI6^pos^ SPP1^pos^	–	–	↑ leukocyte-rich RA	Mass cytometry: CD11C+ CD38+	Not specified	
(53)	Human	MerTK^pos^ CD206^pos^	TREM2^pos^ FOLR2^pos^	TREM2^pos^ TimD4^pos^ CD163^high^	↑ Healthy donors ↑ RA remission ↓ Active RA ↓Treatment naive	–	MerTK^pos^ TREM2^pos^ cells form a neat lining layer in healthy and RA remission synovium. The cells are dispersed in active RA. TREM2^high^ macrophages are homologs of mouse TREM2^pos^ CX3CR1^pos^ lining layer cells.	Gene expression suggests a role for microbe, apoptotic cell and oxysterol clearance as well as restraining inflammation. TREM2^high^ cells express tight junctional protein genes suggesting barrier functions.
				TREM2^low^	↑ Healthy ↑ RA remission ↑ UPA ↓ Active RA	–	–
			FOLR2^high^ TREM2^neg^	D2^pos^	Similar proportions in healthy and RA tissues.	–	Not specified	Possible equivalent to mouse M-CSF-driven *in situ* precursors of resident macrophages.
				LYVE1^pos^	↑ Healthy ↑ RA remission ↓ Active RA ↓ Treatment naive	–	Localised to lining layer in healthy and remission RA. Localised round sublining layer blood vessels in active RA.	Express genes related to collagen turnover, antiprotease enzymes, coagulation factors and regulators of VEGF.
				ICAM1^pos^	Similar proportions in healthy and RA tissues.	–	Not specified	High expression of proinflammatory cytokine genes e.g., TNF.
		MerTK^neg^ CD206^neg^	HLA^high^ CD48^pos^	ISG15^pos^	↑ Active RA	–	Not specified	–
				CLEC10A^pos^	Similar proportions in healthy and RA tissues identified by SCRNA-seq, but flow cytometry suggests increases in active RA.	–	Exclusively located in the sublining layer, located adjacent to TREM2^pos^ macrophages in all samples.	Enriched in antigen-presentation pathway genes, DC markers and transcription factors suggesting this is a tissue-resident antigen presenting population. Has high HBEGF expression, shown to promote fibroblast invasiveness.
			CD48^pos^	S100A12^pos^	↑ Active RA↑ Treatment naïve	–	Located in sublining layer	Abundance of alarmins acting as chemoattractants for neutrophils and monocyte/fibroblast production of TNF and IL-6.
				SPP1^pos^	↑ Active RA ↑ Treatment naïve ↓RA remission	–	Located in sublining layer	Osteopontin is highly expressed in this cluster and has proinflammatory and bone reabsorbing properties.
(51)	Mouse	CD45+ CD11b+ Ly6G+ CX3CR1^pos^ lining macrophages	–	–	Spatial location and morphology alter upon inflammatory arthritis onset	SC-RNA-seq: TREM2^pos^ VSIG4^pos^ Sparc^pos^	Membrane-forming lining macrophages located between synovial capillary network and intra-articular space.	Express tight junctional proteins e.g., ZO-1, claudin-5 and JAM-1.
		CD45+ CD11b+ Ly6G+ CX3CR1^neg^ interstitial macrophages	MHCII^pos^	–	–	SC-RNA-seq: H2-EB1^pos^ H2-AB1^pos^	Located within synovial interstitium	Proliferate to contribute to the pool of CX3CR1^pos^ lining macrophages. Proliferation is enhanced during arthritis onset.
			RELM-α^pos^	–	–	SC-RNA-seq: MRC1^pos^ CD163^pos^ CCL8^pos^ CCL7^pos^		
			AQP1^pos^	–	–	SC-RNA-seq: FXYD2^pos^ LYVE1^pos^		

## Synovial Macrophage Subsets in OA

Macrophages in OA are not completely understood and most studies to date refer to macrophages as M1/M2, as comprehensively reviewed by Fernandes and colleagues (54). M1 macrophages in OA are linked with destructive processes: down regulation of collagen type II and aggrecan synthesis, and upregulation of enzymes such as MMP-1, -3, -9 and -13 (55). In comparison, there is murine evidence that “tissue repairing” M2 macrophage associated cytokines IL-4 and IL-10 are induced with moderate physical activity within the OA synovium, potentially promoting a protective environment (56). Macrophage-related chemokine CCL2, produced in response to inflammatory stimuli by chondrocytes, and its receptor CCR2 has also been noted. Depletion of CCL2/CCR2 is associated with decreased pain severity and older knockout mice show reduced structural disease after joint de-stabilisation (57). The upstream inducer of CCL2, TGF-α has been identified as a possible gene candidate for determining human OA risk and cartilage thickness. TGF-α inhibition in models also shows reduced structural disease, making this an interesting macrophage-related therapeutic target (58). In other mouse studies, contradictory results are reported. Macrophage depletion has been shown to reduce OA symptoms such as osteophyte formation in some cases, but in others increased synovitis can be seen due to CD3^+^ T cell and neutrophil infiltration (59, 60). Discrepancies of macrophage manipulation in mouse models however could relate to the mechanism of disease onset used e.g., obesity, surgical etc., something which may suggest the presence of possible OA phenotypes.

With new knowledge of RA synovial macrophage subsets emerging that goes beyond the classical M1/M2 concept, there is further potential to generate new therapeutic strategies to promote the resolution of synovitis in OA, specifically targeting patients that would likely benefit most. Whether the same cellular populations and mechanisms exist in OA remains unclear and is currently under investigation. Nevertheless, the first study to explore and characterise the cellular and transcriptional heterogeneity on a single cell level in matched synovial and cartilage from OA patients was recently published by the Kraus laboratory (61). Here the authors identify twelve synovial cellular populations, including two distinct macrophage populations, and show that key OA mediators (TNF, IL-6 and IL-1β) are released into the joint space *via* HLA-DRA+ macrophages and DCs. Cytokine expression was 25-fold higher within the synovium compared to damaged cartilage areas and no cytokine was exclusively expressed by chondrocytes themselves. This emphasises the possible crosstalk between joint tissues in OA development. Such cytokine upregulation in the synovium may have synergistic effects on signalling pathways within other joint tissues to increase inflammation and in turn promote cartilage breakdown (62). This study again displays a potential for tissue specific targeting of pathogenic molecules or cells within the synovium itself in order to treat OA (61).

A recent study from our own laboratory identified distinct human knee OA endotypes (where insights of the pathogenic mechanism of disease are given) based on gene expression profiles of synovial macrophages. One of these endotypes displayed increased numbers of synovial CD14+ macrophages that closely aligned with synovial macrophages from inflammatory arthritis patients and displayed a cell proliferation signature and high Ki67 expression (45). However, whether this finding is in any way comparable with Ki67-expressing CX_3_CR_1_- macrophages in mice remains to be answered. The discovery of multiple synovial macrophage subsets may help to explain the contradicting results derived from *in vivo* macrophage-depletion studies of OA, and it is thought that macrophage subset identification could be used to aid the stratification of patients for treatment. Understanding the impact of specific synovial macrophage subsets is a research priority, with fundamental questions remaining. Do the same macrophage subsets exist in OA as in RA? Do macrophage subsets differ between OA disease stages? Do particular macrophage subsets associate with clinical symptoms? And importantly, what other cells within the synovial environment do macrophage subsets communicate with or influence? Unearthing such information could prove crucial for understanding OA pathogenesis and importantly reveal new therapeutic targets. Identification of several macrophage subsets within joint tissue truly advocates for an alternative assessment of how this cellular population is involved in OA.

## Future Directions

New directions for OA research are imperative as clinical trials for disease-modifying treatments thus far have been largely disappointing. Disease heterogeneity often is suggested as a possible explanation. In 2016 Dell’Isola and colleagues provided evidence for the existence of six major OA clinical phenotypes (where observable traits are used to define disease clusters), reporting that 84% of subjects across twenty-four studies could be classified in this manner (63). 12% of OA patients could be classified into an “inflammatory” phenotype, whilst the others were characterised into chronic pain, metabolic syndrome, bone and cartilage metabolism, mechanical overload and minimal disease phenotypes. Regardless of such classifications, trials of anti-IL-1 agents that specifically focused on patients with synovitis (the inflammatory phenotype) still resulted in limited improvement in pain scores and synovial inflammation (64). This implies factors other than IL-1 are at play in this patient subgroup. The ability to further classify patients more effectively could significantly transform and enhance OA clinical trial efficiency. Such approaches have already been applied in other settings such as in RA and asthma, as a method for identifying “clinicopathobiologic clusters” (65–67). By selecting patients based on particular OA molecular features, such as signalling pathways or other distinct molecular mechanisms as opposed to only by clinical phenotype, such as the presence of synovitis, patient subgroups most likely to benefit from particular therapies may be more easily identified. Of course, revealing molecular endotypes of OA is an extremely complex task. Advancements in imaging techniques, identification of novel OA biomarkers and increased knowledge of cellular communications (like that of macrophage subsets) within joint tissues will be of great importance. Ultimately, this approach could facilitate the development of better treatment strategies for OA patients.

## Author Contributions

AT and CH conceived the idea of the manuscript and performed literature searches. AT wrote the manuscript. CH reviewed and edited the manuscript. All authors contributed to the article and approved the submitted version.

## Funding

This work was funded by the JGW Patterson Foundation.

## Conflict of Interest

The authors declare that the research was conducted in the absence of any commercial or financial relationships that could be construed as a potential conflict of interest.
